# Effect of thermal radiation on convective heat transfer in MHD boundary layer Carreau fluid with chemical reaction

**DOI:** 10.1038/s41598-023-31151-4

**Published:** 2023-03-13

**Authors:** Syed Amir Ghazi Ali Shah, Ali Hassan, Hanen Karamti, Abdullah Alhushaybari, Sayed M. Eldin, Ahmed M. Galal

**Affiliations:** 1 Department of Mathematics, Capital University of Science and Technology, Islamabad, Pakistan; 2 Department of Mathematics, University of Gujrat, Gujrat, 50700 Pakistan; 3 Department of Computer Sciences, College of Computer and Information Sciences, Princess Nourah bint Abdulrahman University, P.O. Box 84428, 11671 Riyadh, Saudi Arabia; 4 Department of Mathematics, College of Science, Taif University, P.O. Box 11099, 21944 Taif, Saudi Arabia; 5 Center of Research, Faculty of Engineering, Future University in Egypt, New Cairo, 11835 Egypt; 6 Department of Mechanical Engineering, College of Engineering in Wadi Alddawasir, Prince Sattam Bin Abdulaziz University, Wadi Alddawasir, Saudi Arabia; 7 Production Engineering and Mechanical Design Department, Faculty of Engineering, Mansoura University, P.O 35516, Mansoura, Egypt

**Keywords:** Applied mathematics, Mechanical engineering

## Abstract

The temperature dependent thermophysical fluid properties have numerous aspects in different industries and engineering processes in which heat transmission is based on fluid flow. For such heat transmission processes, heat transmission system is highly fluctuated with variation of viscosity. Thus, the aim of this study is to investigate the transfer of heat in magnetized Carreau fluid with chemical reaction and under influence of thermal radiation over nonlinear stretching/shrinking surface. Additionally, we have incorporated variable heat dependent thermophysical properties to analyze the heat transfer in magnetized Carreau fluid. Set of flow governing non linear PDE’s are obtained using Carreau fluid tensor and boundary layer approximation (BLA) theory. Dimensionless set of ODE’s are obtained using suitable similarity transforms. Shooting method in conjunction with Newton's method have been utilized to solve the problem. It is noted that when stretching $B\ge 0$ is significant with strictly increasing mass suction $S$ shear stress rate increase with minor levels and sharp increase has been observed in Nusselt number, whereas in shrinking case $B<0$ shear stress and heat transfer coefficient values are improved raising the value of $S$ mass suction. Further, raising the values of power law index $n$ produce reduced skin friction over stretching surface $B\ge 0$ while skin friction dramatically enhance in shrinking case $B<0$. It is observed that raising the non-linearity $m$ values for stretching or shrinking, skin friction and Nusselt number considerably improved. Moreover, computational outcomes of the study are validated with already published previous results and the results obtained in this study are found in good agreement.

## Introduction

In recent decades heat transfer enhancement has been a interesting area due to its numerous applications in industries and engineering. Nanofluid are formed when a nano-particle is dispersed in a base fluid namely; water, oils and ethylene glycol. Thermal conductivity of nanofluid is considerably high than convection fluids. The innovation of a new concept hybrid nanofluid, thermal conductivity of poor performing nanofluids has significantly improved. The application of nanofluids includes drilling, electronic cooling, thermal storage, heat and cooling of buildings and microwave tubes^1^. Nanofluids have plethora of applications in bio-medical such as drug delivery, cancer therapeutics, cryosurgery, sensing and imaging^2^. Nanofluids have numerous applications in thermal engineering namely; collectors and solar water heaters, solar cells and solar stills^3^.

Goud et al.^4^ studied MHD effect on Non Newtonian Casson model subjected to chemical over exponential surface with chemical reaction and heat transfer. Goud and Reddy^5^ discussed unsteady heat absorption effect with radiation and heat transfer in flow along infinite vertical plate. Arshad et al.^6^ explored Maxwell fluid with chemical reaction over infinte surface embedded in porous medium for heat enhancement. Reddy et al.^7^ investigated heat transfer over stretching cylinder under generation/absorption effects. Hassan et al.^8^ examined heat transfer coefficient using hybrid nanofluids using multiple base fluid under different thermal radiation aspects. Kavya et al.^9^ demonstrated magnetic hybrid nano-particles over stretching/shrinking cylinder. Reddy et al.^10^ studied hybrid nanofluid with magnetic field in a irregular vertical channel. Shah et al.^11^ discussed Non-Newtonian Carreau fluid with convective conduction to investigate the heat transfer with variable thermophysical fluid properties. Goud et al.^12^ inspected magneto-hydrodynamic convection in a flow under Soret effect when viscous dissipation is significant. Waqas et al.^13^ explored hybrid nanofluids with silver and gold nano-particles for heat enhancement in a stenosed artery. Some recent studied on heat transfer using nano and hybrid nanofluids^14–18^.

Chemical reaction occur when two components collide to form a product in the presence of some assisting force namely catalyst. Chemical reactions are of two kinds namely; reversible and irreversible chemical reactions. Chemical reactions which can not be reversed are known as irreversible chemical reactions whereas those chemical reaction which can be reversed are known as reversible chemical reactions. Chemical reaction assist the nano-particle movement in the flow and heat transfer^19^. Additionally, significance of first order ad higher chemical reaction on the motile microorganism migration and heat transmission has been addressed by numerous researcher in recent years. Yanala et al.^20^ studied slip effect on transient laminar flow with ramped temperature and first order chemical reaction. Goud et al.^21^ discussed Non Newtonian Casson model with joule heating and chemical reaction. Hosseinzadeh et al.^22^ examined nonlinear radiation effect on Maxwell fluid with convective condition subjected to chemical reaction. Khan et al.^23^ explored third grade magneto-hydrodynamic flow with variable reactive index with chemical reaction effect. Mythili and Sivaraj^24^ investigated non uniform heat source and sink effect with chemical reaction on Non Newtonian Casson fluid flow. Malik and Rehman^25^ elaborated effect of second order chemical with heat generation on the free convective flow over inclined surface. Some recent studies with second and higher order chemical reaction effects on different flow regimes^26–29^.

Radiation is characterized as distribution of thermal electromagnetic particles through a medium. Thermal radiation therapy is a kind of treatment employed by doctors in a pathological situation which involve transmission of heat below the skin friction into the muscles and tissues. It has been observed that electromagnetic heat namely; shortwaves and microwaves send heat upto 2 inches into the tissues and muscles^30,31^. Reddy et al.^32^ discussed ramped plate temperature and heat absorption under thermal radiation effect for magneto-hydrodynamic boundary layer flow. Ge-JiLe et al.^33^ analyzed the effect of radiation on magnetic fluid with Brownian and Thermophoresis particle diffusion. Hassan et al.^34^ explored the prescribed wall temperature case with hybrid nano-particle under thermal radiation. Hussain et al.^35^ demonstrated heat transmission in hybrid flow of single and multi-wall carbon nanotube with radiation impacts. Reddy et al.^36^ elaborated heat transfer in Casson nanofluid with viscous dissipation effect with particle movement under the impact of thermal radiation. Recently, constructive studies have been carried out to analyze thermal radiation impact with comprehensive flow regimes^37–41^.

The variable thermophysical fluid properties play significant role in heat transfer enhancement of fluid. The temperature dependent thermal conductivity and temperature dependent viscosity has significant impact on heat transfer. Animasaun and Sandeep^42^ investigated variable thermal conductivity and viscosity dependent on concentration in nanofluid flow. Salahuddin and Awais^43^ examined Carreau-Cross fluid comparatively with variable thermal conductivity and thermal radiation. Abbas et al.^44^ explored magneto-hydrodynamic Carreau flow with homotopy method with variable fluid properties. Khan et al.^45^ examined Carreau fluid surface with variable thickness using Keller Box method. Nalivale et al.^46^ studied natural convective flow with viscous dissipation and thermal radiation effects. Megahed et al.^47^ described Carreau fluid with heat flux and variable thermal conductivity under thermal radiation impact. Irfan et al.^48^ studied heat source/sink and generalized Fourier’s law under variable thermal conductivity influence in Carreau fluid flow. Waqas et al.^49^ discussed variable fluid properties effect on Carreau fluid with mixed convection in stagnation point flow.

In the above conducted literature review plethora of researchers had discussed the nanofluids with externally applied effects for heat transfer enhancement. Animasaun and Sandeep^42^ used variable thermal conductivity and variable visocity dependent on concentration to discuss the nanofluid flow over stretched surface. They ignored thermal and chemical impacts in their study. While, Salahuddin and Awais^43^ investigated Cross and Carreau model comparatively employing only variable thermal conductivity with thermal radiation. Abbas^44^ used analytical methodology to study the impact of variable fluid properties effect on Carreau fluid with MHD effect ignoring the chemical transportation. Khan^45^ elaborated the Carreau fluid over surface of variable thickness using Keller Box method in the absence of any variable thermophysical fluid property. Megahed^47^ studied Carreau fluid with heat flux and radiation effect using only variable thermal conductivity model and in the absence of variable viscosity. Irfan^48^ discussed variable thermal conductivity with generalized Fourier law and Waqas^49^ used both variable thermal conductivity and viscosity to examine Carreau flow model in stagnation point regime.

The purpose of this study is to explore the impact variable heat dependent fluid properties such as thermal conductivity and viscosity on radiative heat transfer in magnetized Carreau fluid model subjected to first order chemical reaction. The novelty of the current article is to examine heated boundary condition, chemical reaction and Rosseland radiation approximation effect on magnetized Non Newtonian Carreau fluid. Furthermore, Abbas^44^ used homotopy analysis method, Khan^45^ employed Keller box method to dicuss Carreau fluid, Irfan^48^ computed Carreau fluid with BVP-4c, Waqas^49^ explored series solution of Carreau fluid. In this work, we have used shooting method with Newton’s method to solve the Carreau fluid flow. The impact of varying fluid parameters on different study profiles is obtained and presented graphically. Shear stress and heat transfer coefficient are presented in tabulated data set.

## Formulation of problem

### Problem description

Let us consider flow of in-compressible magnetized Non-Newtonian Carreau fluid flow under the effect of thermal radiation and subjected to first order chemical reaction over a stretched surface with heated boundary. The surface has been stretched with the ${u}_{w}=a{x}^{m}$ stretching velocity in horizontal direction. Where, $m$ in surface stretching velocity denote the non linearity parameter. Whereas the surface as ambient condition of temperature and concentration are represented with well known notations ${T}_{w},{T}_{\infty } and {C}_{w}, {C}_{\infty }$, respectively. Thus the general flow governing equations are given as follows:

Continuity equation^11,44,44^

1 $$\frac{\partial u}{{\partial x}} + \frac{\partial v}{{\partial y}} = 0.$$

Momentum equation^11,44,47^

2 $$u\frac{\partial u}{{\partial x}} + v\frac{\partial u}{{\partial y}} = \frac{1}{\rho }\frac{\partial }{\partial y}\left[ {\mu \frac{\partial u}{{\partial y}}} \right] + 3v_{f} \frac{n - 1}{2}\Gamma^{2} \left( {\frac{\partial u}{{\partial y}}} \right)^{2} \frac{{\partial^{2} u}}{{\partial y^{2} }} + \frac{\sigma }{\rho }J^{2} \left( {u_{e} - u} \right) + u_{e} \frac{{\partial u_{e} }}{\partial x}.$$

Heat equation

3 $$u\frac{\partial T}{{\partial x}} + v\frac{\partial T}{{\partial y}} = \frac{{k_{f} }}{{\left( {\rho c_{p} } \right)_{f} }}\left[ {\frac{{\partial^{2} T}}{{\partial y^{2} }}} \right] - \frac{1}{{\left( {\rho c_{p} } \right)_{f} }}\frac{{\partial \left( {q_{r} } \right)}}{\partial y}.$$

Species transfer equation^11,44^

4 $$u\frac{\partial C}{{\partial x}} + v\frac{\partial C}{{\partial y}} = D\frac{{\partial^{2} C}}{{\partial^{2} y}} - K_{r} (C - C_{\infty } ).$$

All the terminologies have been given in the nomenclature section so we must omit elaborating them here. In the above Eqs. (1–4), $u,v$ are components of the velocity vector, $\mu$ denote dynamic viscosity, $n$ describe the power law index, is electrical conductivity, $\rho$ denotes the density, ${u}_{e}$ represent some external velocity, $j$ denote the magnetization force, ${k}_{f}$ is temperature, is thermal conductivity, $(\rho {C}_{p})$ describe the specific heat capacity, $C$ denote the species profile, $D$ describe the thermal diffusivity, ${K}_{r}$ and represent the first order chemical reaction. Further, as we inspecting the Carreau fluid with variable fluid properties. Therefore, the relations used in this study to incorporate the effect of variable heat dependent fluid properties are given in following study^11^:

5 $$\mu \left( T \right) = \mu^{*} \left[ {N_{1} - h_{1} \left( {T_{\infty } - T} \right)} \right],k_{f} \left( T \right) = k_{f} *\left[ {N_{2} - h_{2} \left( {T_{\infty } - T} \right)} \right].$$

The boundaries associated with above flow governing model are given as^11^:

6 $$\begin{aligned} & u = u_{w} \left( x \right) = ax^{m} ,v = v_{w} \left( x \right),\frac{\partial T}{{\partial y}} = - \frac{{q_{w} \left( x \right)}}{{k_{f} }},C = C_{w} \quad at\quad y \to 0, \\ & u \to u_{e} \left( x \right) = bx^{m} ,T \to T_{\infty } ,C \to C_{\infty } \quad at\quad y \to \infty . \\ \end{aligned}$$

**Figure 1:**
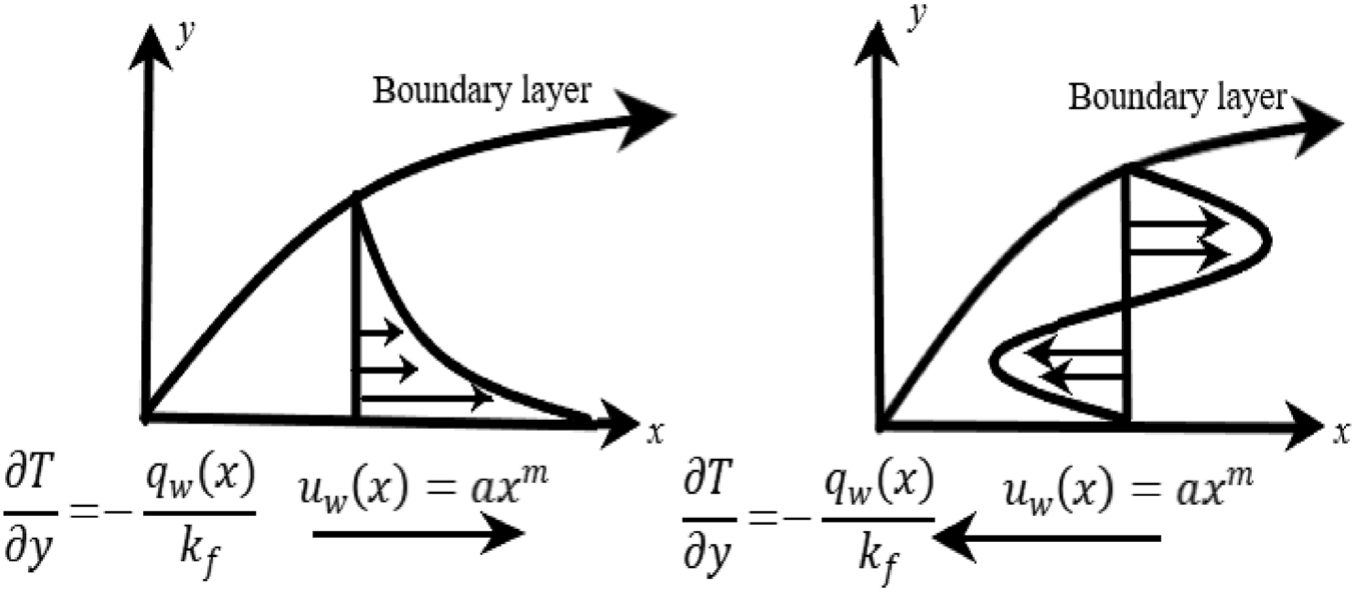
Representation of physical model.

${\mu }^{*}$ and ${{k}_{f}}^{*}$ in the preceding equation describe the effect of heat dependent viscosity and thermal conductivity. whereas ${h}_{1}, {h}_{2}, {N}_{1} and {N}_{2}$ are some positive constants. In addition, the values ${N}_{1} and {N}_{2}$ are set to 1. Figure 1 show the configuration and coordinate system of present problem.

### Similarity transform and dimensionless flow equations

Suitable similarity transforms used in this study are reported by Shah^11^. We are employing same similarity transforms to convert dimensional flow equations into non dimensionless form. The similarity transform are as follow:

7 $$\theta \left( \eta \right) = \frac{{T - T_{w} }}{{\left( {T_{\infty } - T_{w} } \right)}},\phi \left( \eta \right) = \frac{{T - T_{w} }}{{\left( {T_{\infty } - T_{w} } \right)}},\varphi = \left( {bv} \right)^{\frac{1}{2}} x^{{\frac{m + 1}{2}}} f\left( \xi \right),\xi = \left( \frac{b}{v} \right)^{\frac{1}{2}} yx^{{\frac{m - 1}{2}}} .$$

Stream function is denoted by $\varphi$. The dimensionless flow governing equations are given as follow:

8 $$\begin{aligned} & f^{\prime\prime\prime}\left( \xi \right) + m\left( {1 - f^{{\prime}{2}} \left( \xi \right)} \right) + \frac{m + 1}{2}f\left( \xi \right)f^{\prime\prime}\left( \xi \right) + \xi f^{\prime\prime\prime}\left( \xi \right) - \xi \left( {f^{\prime\prime}\left( \xi \right)\theta^{\prime}\left( \xi \right) + f^{\prime\prime\prime}\left( \xi \right)\theta \left( \xi \right)} \right) \\ & \quad + \frac{3}{2}\left( {n - 1} \right)We^{2} f^{{\prime\prime}{2}} \left( \xi \right)f^{\prime\prime\prime}\left( \xi \right)M^{2} \left( {1 - f^{\prime}\left( \xi \right)} \right) = 0, \\ \end{aligned}$$

9 $$\theta^{\prime\prime}\left( \xi \right) + Pr\frac{{\frac{m + 1}{2}}}{{1 - \frac{4}{3}R}}\theta^{\prime}\left( \xi \right)f\left( \xi \right) + \frac{\varepsilon }{{1 - \frac{4}{3}R}}\left( {\theta \left( \xi \right)\theta^{\prime\prime}\left( \xi \right) + \theta^{{\prime}{2}} \left( \xi \right)} \right),$$

10 $$\phi^{\prime\prime}\left( \xi \right) + Le\frac{m + 1}{2}\phi^{\prime}\left( \xi \right)f\left( \xi \right) + Le\frac{m + 1}{2}\phi \left( \xi \right)\gamma .$$

The associated boundaries are given as:

11 $$\begin{aligned} & f\left( 0 \right) = S,f^{\prime}\left( 0 \right) = B,\theta^{\prime}\left( 0 \right) = 1,\phi \left( 0 \right) = 1,\quad at\quad \xi \to 0 \\ & f^{\prime}\left( \xi \right) \to 1,\theta \left( \xi \right) \to 0,\phi \left( \xi \right) \to 0,\quad at\quad \xi \to \infty . \\ \end{aligned}$$

In the above equations ${\mathrm{We}}^{2}=\frac{{\Gamma }^{2}{b}^{3}{x}^{3m-1}}{{\upupsilon }_{\mathrm{f}}}$ denotes Weissenberg number, ${\mathrm{M}}^{2}=\frac{\upsigma {\mathrm{J}}^{2}}{\mathrm{\rho b}}$ is magnetic field parameter, $\mathrm{S}=\frac{-2{v}_{w}}{(bv{)}^{1/2}(m+1) {x}^{\frac{m-1}{2}}}$ is suction/injection parameter, $B=\frac{a}{b}$ is stretching ratio, Prandtl number is given as $Pr=\frac{\mu {C}_{p}}{{k}_{f}}$, variable heat dependent viscosity is denoted by $\upxi = {h}_{1}({T}_{\infty }-{T}_{w})$, variable heat dependent thermal conductivity is represented by $\upepsilon = {h}_{2}({T}_{\infty }-{T}_{w})$, $R=\frac{4{\sigma }^{*}{T}_{0}^{3}}{{k}^{*}{k}_{f}}$ is thermal radiation parameter,$\gamma =\frac{2{K}_{r}}{b(m+1){x}^{m-1}}$ is chemical reaction parameter and $Le=\frac{{\nu }_{f}}{D}$ Lewis Number.

## Methodology of numerical solution

Abbas^44^ used homotopy analysis method, Khan^45^ employed Keller box method to dicuss Carreau flui, Irfan^48^ computed Carreau fluid with BVP-4c, Waqas^49^ explored series solution of Carreau fluid. In this work, we have used shooting method with Newton’s method to solve the Carreau fluid flow. The procedure of shooting method is elaborated below:

12 $$\begin{aligned} & f = Y_{1} ,f^{\prime} = Y^{\prime}_{1} = Y_{2} ,f^{\prime\prime} = Y^{\prime\prime}_{2} = Y_{3} ,f^{\prime\prime\prime} = Y^{\prime}_{3} ,\theta = Y_{4} ,\theta^{\prime} = Y^{\prime}_{4} = Y_{5} ,\theta^{\prime\prime} = Y^{\prime}_{5} , \\ & \phi = Z_{1} ,\phi^{\prime} = Z^{\prime}_{1} = Z_{2} ,\phi^{\prime\prime} = Z^{\prime}_{2} . \\ \end{aligned}$$

The obtained first order ODE’s after using the notation (12) are given as follow:

13 $$Y^{\prime}_{1} = Y_{2} ,$$

14 $$Y^{\prime}_{2} = Y_{3} ,$$

15 $$Y^{\prime}_{3} = \left[ {\frac{{\left( { - m(1 - Y^{2}_{2} } \right) - \frac{m + 1}{2}Y_{1} Y_{3} + \xi Y_{3} Y_{5} - M^{2} \left( {1 - Y_{2} } \right)}}{{1 + \xi - \xi Y_{4} + \frac{3}{2}\left( {n - 1} \right)We^{2} Y_{3}^{2} }}} \right],$$

16 $$Y^{\prime}_{4} = Y_{5} ,$$

17 $$Y^{\prime}_{5} = \left[ {\frac{{\left( {1 - \frac{4}{3}R} \right)\left( { - \Pr \frac{m + 1}{2}Y_{1} Y_{5} - \varepsilon Y^{2}_{5} } \right)}}{{1 + \varepsilon Y_{4} }}} \right],$$

18 $$Z^{\prime}_{1} = Z_{2} ,$$

19 $$Z^{\prime}_{2} = - \frac{m + 1}{2}LefZ_{2} - \frac{m + 1}{2}\gamma LeZ_{1} .$$

Suitable boundary conditions are given as:

20 $$Y_{1} \left( 0 \right) = S,Y_{2} \left( 0 \right) = B,Y_{3} \left( 0 \right) = r,Y_{4} \left( 0 \right) = q,Y_{5} \left( 0 \right) = 1,Z_{1} \left( 0 \right) = 1,Z_{2} \left( 0 \right) = Q.$$

The missing conditions $r,q and Q$ assumed to validate the below relations:

21 $$Y_{2} \left( {\xi ,r,q} \right) = 1,Y_{2} \left( {\xi ,r,q} \right) = 0,Z_{1} \left( {\xi ,r,q} \right) = 0.$$

The stopping criterion is given as:

22 $$\max \left\{ {\left| {Y_{2} \left( {\xi_{\infty } } \right) - 1} \right|,\left| {Y_{4} \left( {\xi_{\infty } } \right)} \right|} \right\} < \delta ,and\left| {Z_{1} \left( {\xi_{\infty } ,Q} \right)} \right| < \delta .$$

## Results and discussion

In this section the effect of different study parameters such as stretching ratio, variable thermal conductivity, variable viscosity, magnetic field, Lewis number, Weissenberg number, suction/blowing, and Prandtl number is discussed on the velocity, temperature and concentration profiles. Additionally, the shear stress rates and Nusselt number under influence of different parameter are also given in tabulated data set.

Through diagrams and figures, the physical impact of major parameters on skin friction, Nusselt number, and Sherwood number has been discussed. In the present survey, the shooting method has been opted for reproducing the values of $C{f}_{x}$ and $N{u}_{x}$. The results presented in Tables 1, 2, 3 discuss the impact of mass suction, power law index and non linearity influence combined with magnetization force illustrate on $C{f}_{x}$ and $N{u}_{x}$.

**Table 1:** Numeric results of skin friction and heat transfer coefficient under influence of stretching ratio and strong mass suction impact.

Fixed values			
$$M=0.5,n=0.5,m=0.3, We=0.3, \xi =0.5, \epsilon =0.5, Pr=0.7, R=-0.2$$			
*B*	*S*	$C{f}_{x}$	$N{u}_{x}$
0	5	2.9150	3.7910
0	5.5	3.0239	4.1949
0	6	3.1283	4.6006
0	6.5	3.2286	5.0078
0	7	3.3250	5.4160
0	7.5	3.4283	5.8006
0	8	3.5286	6.2078
0	8.5	3.6250	6.6160
0	9	3.7240	7.1160
− 3	5	4.6336	3.2377
− 3	5.5	4.8673	3.6922
− 3	6	5.0804	4.1397
− 3	6.5	5.2772	4.5819
− 3	7	5.4604	5.0202
− 3	7.5	5.6150	5.5910
− 3	8	5.8336	6.0377
− 3	8.5	6.0673	6.5922

**Table 2:** Numeric results of skin friction and heat transfer coefficient under influence of stretching ratio and power law index impact.

Fixed values			
$$M=0.5,S=5,m=2, We=0.3, \xi =0.5, \epsilon =0.5, Pr=0.7, R=-0.2$$			
*B*	*n*	$C{f}_{x}$	$N{u}_{x}$
2	5	− 3.0564	3.9932
2	6	− 2.8924	3.9954
2	7	− 2.7628	3.9973
2	8	− 2.6564	3.9989
2	9	− 2.5666	4.0004
− 2	5	4.4452	3.4641
− 2	6	4.1541	3.4529
− 2	7	3.9285	3.4436
− 2	8	3.7460	3.4358
− 2	9	3.5939	3.4290

**Table 3:** Numeric results of skin friction and heat transfer coefficient under influence of stretching ratio and non linearity effect.

Fixed values			
$$M=0.5,S=5,n=5, We=0.3, \xi =0.5, \epsilon =0.5, Pr=0.7, R=-0.2$$			
*B*	*m*	$C{f}_{x}$	$N{u}_{x}$
0	7	4.4039	10.6706
0	7.5	4.5109	11.3597
0	8	4.6136	12.0488
0	8.5	4.7236	13.0388
0	9	4.8074	13.4275
0	10	4.9880	14.8064
− 3	7	6.8370	10.0829
− 3	7.5	6.9991	10.7710
− 3	8	7.1548	11.4594
− 3	8.5	7.2048	12.4194
− 3	9	7.4495	12.8367
− 3	10	7.7248	14.2147

**Figure 2:**
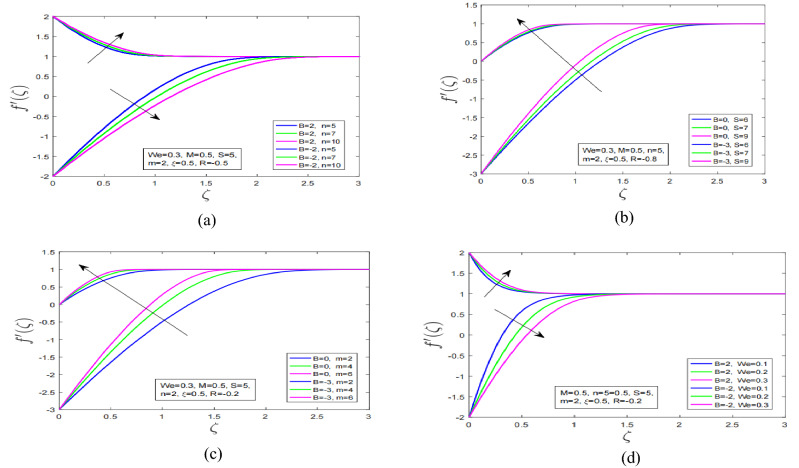
(**a**) Power law index effect on ${f}^{^{\prime}}$. (**b**) Suction impact on ${f}^{^{\prime}}$. (**c**) Non-linearity impression on ${f}^{^{\prime}}$. (**d**) Weissenberg number outcome on ${f}^{^{\prime}}$.

Figure 2a shows the combine impact of power law index and magnetization effect on Carreau fluid over stretching and shrinking surface. It is observed that with increment in power law index velocity of Carreau fluid has increased for stretching surface whereas when power law index in augmented with magnetic field for shrinking surface the motion of Carreau fluid has decreased. Additionally, momentum boundary layer has expanded under power law index and magnetic force for shrinking surface. Figure 2b depicts influence of mass suction effect with magnetic field. It is worth noting that over the stretching and shrinking surface the motion of Carreau fluid has increased. Furthermore, interestingly momentum layer associated with shrinking surface has expanded under high impact mass suction effect. Figure 2c describe the impression of non linearity in the presence of strong magnetization force on velocity profile. When the non linearity in either stretching or shrinking is incremented motion profile of Carreau fluid show an increasing behaviour. It is observed that with increment in non linearity the momentum layer has expanded more rapidly for shrinking surface as compared stretching surface.

**Figure 3:**
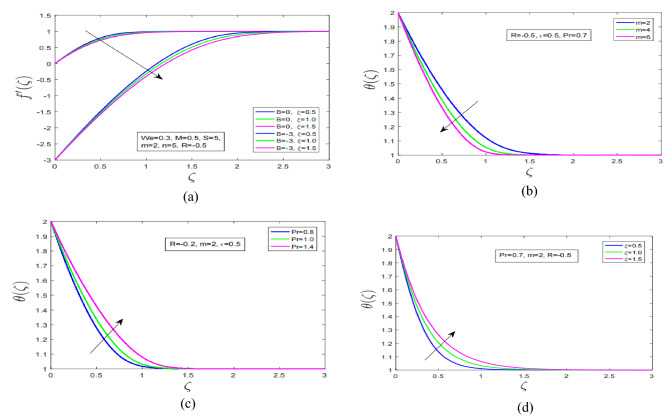
(**a**) Varying viscosity effect on ${f}^{^{\prime}}$. (**b**) Non-linearity impact on $\theta$. (**c**) Prandtl number influence on $\theta$. (**d**) Varying viscosity impression on $\theta$

Figure 2d discuss the impact of Weissenberg number on motion profile of Carreau fluid in the presence of strong magnetic force over the stretching and shrinking sheet. Carreau fluid velocity has decreased over stretching surface with augmentation in Weissenberg number. On the other hand it is observed that increment in Weissenberg number and magnetic field has increased Carreau fluid motion profile over shrinking surface. Figure 3a illustrate the effect of variable viscosity parameter on Carreau fluid motion profile over stretching and shrinking surface. It is discovered that with increment in viscosity of Carreau fluid the fluid motion over stretching and shrinking surfaces has dramatically decreased. This effect occurs due to enhancement in boundary layer thickness of Carreau fluid with increment in viscosity of Carreau fluid. Additionally, velocity profile has decreased under combined increment in variable viscosity and magnetic force. The resistive force generated in vicinity of boundary layer due to applied high magnetic field in boundary layer has reduced the motion profile of velocity profile.

Figure 3b shows the impact of non linearity parameter on temperature profile of Carreau fluid over stretching surface. It is observed that with increment in non linearity parameter temperature profile has decreased. Additionally, thermal boundary layer thickness has increased in the presence of high viscosity and thermal conductivity parameter. Figure 3c describe the impact of Prandtl number on temperature profile of Carreau fluid. It is interesting to note here that when Prandtl number is incremented the temperature profile enhance consequently the associated thermal boundary layer has increased. Moreover, it is discovered that with variable viscosity and thermal conductivity the temperature profile has increased under Prandtl number increment. Figure 3d discuss impact of variable viscosity parameter on temperature profile of Carreau fluid. When viscosity of Carreau fluid is increment as a result the thickness of boundary layer increase which consequently decline the associated motion profile and temperature profile. It is worth mentioning here that with increment in viscosity of Carreau fluid thermal boundary layer of fluid contracted due to reduction in thickness of boundary layer.

**Figure 4:**
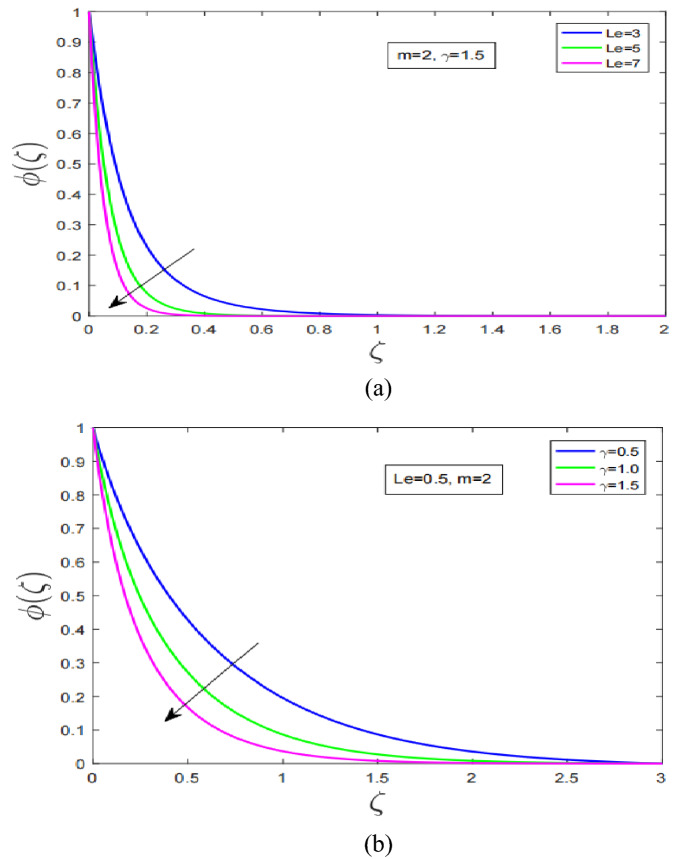
(**a**) Lewis number impact on $\phi$. (**b**) Chemical reaction Influence on $\phi$.

Figure 4a,b discuss concentration profile of Carreau fluid under influence of Lewis number and chemical reaction parameters, respectively. Concentration profile under incremental change in Lewis number has sharply decreased. As a result associated concentration boundary of Carreau fluid has shown rapid contraction (see Fig. 4a). Figure 4b demonstrate impact of chemical reaction parameter on concentration profile. Concentration profile has decreased with increment in chemical reaction parameter. It is worth noting here that concentration layer has expanded with increment in chemical reaction parameter.

Table 1 shows outcomes of shear stress rates and Nusselt number of Carreau fluid under combined impact of magnetic force and mass suction effect. It is interesting to note here that when mass suction effect is incremented in the absence of magnetization force shear stress and heat transfer coefficient have increased. Moreover, with applied strong magnetization force and mass suction effect shear stress and Nusselt further enhance. Table 2 describe the impact of power law index and magnetic field on engineering quantities. Reduced shear stress rates are observed under the increment in power law index and constant applied magnetization. It is also worth mentioning here that shear stress rate increase in Carreau fluid when magnetization force magnitude is turned negative in presence of power law index. Furthermore, Nusselt number has remained consistent with change in attributes of magnetization force. Table 3 illustrate shear stress rate under impact of non linearity parameter. It is observed that by raising the non linearity values shear stress and heat transfer coefficient of Carreau fluid have increased even with high applied magnetization force on Carreau fluid. Table 4 show the agreement between the present outcomes of shear stress and heat transfer coefficient with those of Abbas^44^.

**Table 4:** Comparison of present outcomes of Nusselt number and skin friction with already published results by Abbas^44^.

$$We=0.3, S=0.5, M=0.5, A=3,m=2 and Le=2$$					
$B$	$n$	$C{f}_{x}$		$N{u}_{x}$	
		Abbas ^44^	Present	Abbas ^44^	Present
2	5	− 3.4035	− 2.363179	5.0226	5.072294
	6	− 3.1957	− 2.28582	5.0251	5.073216
	7	− 3.0340	− 2.22025	5.0277	5.0740361
	8	− 2.9030	− 2.1635	5.0302	5.074774
	9	− 2.7935	− 2.113576	5.0302	5.0754468
	10	− 2.7001	− 2.069081	5.0327	5.076063

### Comparison analysis with previous works

In our study if we neglect the Rosseland thermal radiation approximation and first order chemical reaction then our flow governing model will coincide with work of Abbas^44^. Megahed^47^ addressed only temperature dependent thermal conductivity influence on the Carreau fluid. They didn’t consider chemical reaction effect and ignored the species transport in their study. Waqas^49^ also ignored the species transportation phenomenon and convective boundary condition while modelling the flow governing equations. Additionally, they used variable temperature dependent thermal conductivity and viscosity model to investigate the Carreau fluid. Furthermore, Abbas^44^ utilized the analytical method commonly known as homotopy analysis method, whereas in this study we have addressed the Carreau fluid with Shooting method and Newton’s method. If we set the same parametric values as given in Table 1 by Abbas^44^ to obtain the numeric results for the skin friction and Nusselt number, good agreement has been found for Nusselt number between the results achieved by Abbas^44^ and present work. Additionally, raising the chemical reaction expands the thickness of concentration layer and decline species profile.

## Conclusion

In this work, Non-Newtonian Carreau fluid has been numerical investigated in the presence of temperature dependent thermophysical properties over the non-linear stretching/shrinking surface. The Rosseland thermal radiation relation has been taken into account to explore the influence of Rosseland approximation of thermal radiation on Carreau fluid, the fluid has been subjected to first order chemical reaction. Below are the major findings of this study:

1. Motion of Carreau fluid increased with raising the values of mass suction and non-linearity. Whereas decline has been observed when power law index, Wessienberg number variable viscosity values are increased.
2. Prandtl number and viscosity augmentation increase temperature profile whereas non-linearity increment decrease temperature profile.
3. Concentration profile has rapidly decreased by raising values of Lewis number and chemical reaction values. Further, the concentration layer has expanded by raising the values of chemical species transport and chemical reaction.
4. Skin friction and Nusselt number has shown strictly increasing behaviour under raising effect of mass suction for stretching/shrinking.
5. Raising the power law index produce reduced shear rates whereas in the event of shrinking skin friction enhance.
6. Nusselt number has significantly improved by raising the values of non linearity parameter. Stretching has produced reduced skin friction as compared to shrinking surface.

## Data Availability

All data generated or analysed during this study are included in this published article.

## List of symbols

$u,v$: Components of velocity $({\mathrm{ms}}^{-1})$
$${v}_{f}$$: Kinematic viscosity $$({\mathrm{m}}^{2}{\mathrm{s}}^{-1})$$
$T,C$: Dimensional temperature and concentration profile (–)
$${\rho }_{f}$$: Density $$({\mathrm{kg m}}^{-3})$$
${C}_{p}$: Heat capacity at constant pressure $(\mathrm{J k}{\mathrm{g}}^{-1} {\mathrm{K}}^{-1})$
*f*: Velocity profile (–)
$\theta ,\phi$: Temperature and concentration dimensionless profile (–)
*L*$e$: Dimenisonless Lewis number (–)
${h}_{1}, {h}_{2}$: Some positive constant
${N}_{1}, {N}_{2}$: Some positive constant
$S$: Dimenisonless suction/injection parameter (–)
$We$: Dimenisonless Weissenberg number (–)
$BLA$: Boundary layer approximation (–)
$$MHD$$: Magneto-hydrodynamic (–)
$(\rho {c}_{p}{)}_{f}$: Volumetric heat capacity $({\mathrm{JK}}^{-1})$
${T}_{W}, {T}_{\infty }$: Surface temperature and stream temperature, respectively $(\mathrm{K})$
${C}_{W}, {C}_{\infty }$: Surface concentration and ambient concentration, respectively $(\mathrm{K})$
$$\zeta$$: Similarity variable (–)
${\mu (T)}_{f}$: Temperature dependent dynamic viscosity $(\mathrm{kg }{\mathrm{m}}^{-1} {\mathrm{s}}^{-1})$
$$D$$: Mass diffusivity (–)
${q}_{W}$: Surface heat flux (–)
${\mu }^{*},{k}_{f}^{*}$: Viscosity and thermal conductivity effect (–)
*P*$$r$$: Prandtl number (–)
$\xi ,\epsilon$: Variable thermal conductivity and viscosity, respectively (–)
$${Re}_{L}$$: Reynolds number (–)
$M$: Dimenisonless magnetic field parameter (–)
$B$: Stretching/shrinking ratio parameter
${v}_{W}$: Injection and suction velocity (–)

## References

[1] Saidur R, Leong KY, Mohammed HA (2011). A review on applications and challenges of nanofluids. Renew. Sustain. Energy Rev..

[2] Wong KV, De Leon O (2010). Applications of nanofluids: Current and future. Adv. Mech. Eng..

[3] Mahian O, Kianifar A, Kalogirou SA, Pop I, Wongwises S (2013). A review of the applications of nanofluids in solar energy. Int. J. Heat Mass Transf..

[4] Shankar Goud, B., Dharmendar Reddy, Y., & Kenneth Asogwa, K. Chemical reaction, Soret and Dufour impacts on magnetohydrodynamic heat transfer Casson fluid over an exponentially permeable stretching surface with slip effects. *Int. J. Mod. Phys. B.* 2350124 (2022).

[5] Goud BS, Reddy YD (2022). Chemical reaction and Soret effect on an unsteady MHD heat and mass transfer fluid flow along an infinite vertical plate with radiation and heat absorption. J. Indian Chem. Soc..

[6] Arshad M, Hussain A, Hassan A, Shah SAGA, Elkotb MA, Gouadria S (2022). Heat and mass transfer analysis above an unsteady infinite porous surface with chemical reaction. Case Stud. Therm. Eng..

[7] Reddy YD, Goud BS, Nisar KS, Alshahrani B, Mahmoud M, Park C (2023). Heat absorption/generation effect on MHD heat transfer fluid flow along a stretching cylinder with a porous medium. Alex. Eng. J..

[8] Hassan A, Hussain A, Arshad M, Awrejcewicz J, Pawlowski W, Alharbi FM, Karamti H (2022). Heat and mass transport analysis of MHD rotating hybrid nanofluids conveying silver and molybdenum di-sulfide nano-particles under effect of linear and non-linear radiation. Energies.

[9] Kavya S, Nagendramma V, Ahammad NA, Ahmad S, Raju CSK, Shah NA (2022). Magnetic-hybrid nanoparticles with stretching/shrinking cylinder in a suspension of MoS4 and copper nanoparticles. Int. Commun. Heat Mass Transf..

[10] Reddy SRR, Raju CSK, Gunakala SR, Basha HT, Yook SJ (2022). Bio-magnetic pulsatile CuO−Fe_3_O_4_ hybrid nanofluid flow in a vertical irregular channel in a suspension of body acceleration. Int. Commun. Heat Mass Transf..

[11] Shah SAGA, Hassan A, Alsubaie N, Alhushaybari A, Alharbi FM, Galal AM (2022). Convective heat transfer in magneto-hydrodynamic Carreau fluid with temperature dependent viscosity and thermal conductivity. Nanomaterials.

[12] Goud, B. S., Reddy, Y. D., & Asogwa, K. K. Inspection of chemical reaction and viscous dissipation on MHD convection flow over an infinite vertical plate entrenched in porous medium with Soret effect. *Biomass Convers. Biorefinery* 1–12 (2022).

[13] Waqas H, Farooq U, Hassan A, Liu D, Noreen S, Makki R (2023). Numerical and Computational simulation of blood flow on hybrid nanofluid with heat transfer through a stenotic artery: Silver and gold nanoparticles. Results Phys..

[14] Hassan A, Hussain A, Arshad M, Haider Q, Althobaiti A, Elagan SK (2022). Heat transport investigation of hybrid nanofluid (Ag-CuO) porous medium flow: Under magnetic field and Rosseland radiation. Ain Shams Eng. J..

[15] Reddy NN, Reddy YD, Rao VS, Goud BS, Nisar KS (2022). Multiple slip effects on steady MHD flow past a non-isothermal stretching surface in presence of Soret, Dufour with suction/injection. Int. Commun. Heat Mass Transf..

[16] Arshad M, Hassan A (2022). A numerical study on the hybrid nanofluid flow between a permeable rotating system. Eur. Phys. J. Plus.

[17] Reddy YD, Goud BS, Khan MR, Elkotb MA, Galal AM (2022). Transport properties of a hydromagnetic radiative stagnation point flow of a nanofluid across a stretching surface. Case Stud. Therm. Eng..

[18] Mishra P, Kumar D, Dharmendar Reddy Y, Shankar Goud B (2022). Numerical investigation of MHD flow of williamson nanofluid with variable viscosity pasting a wedge within porous media: A non-darcy model approach. Heat Transf..

[19] Hassan, A., Hussain, A., Arshad, M., Gouadria, S., Awrejcewicz, J., Galal, A. M., *et al.* Insight into the significance of viscous dissipation and heat generation/absorption in magneto-hydrodynamic radiative casson fluid flow with first-order chemical reaction. *Front. Phys.* **605** (2022).

[20] Yanala DR, Mella AK, Vempati SR, Goud BS (2021). Influence of slip condition on transient laminar flow over an infinite vertical plate with ramped temperature in the presence of chemical reaction and thermal radiation. Heat Transf..

[21] Goud BS, Reddy YD, Rao VS (2020). Thermal radiation and Joule heating effects on a magnetohydrodynamic Casson nanofluid flow in the presence of chemical reaction through a non-linear inclined porous stretching sheet. J. Nav. Archit. Mar. Eng..

[22] Hosseinzadeh K, Gholinia M, Jafari B, Ghanbarpour A, Olfian H, Ganji DD (2019). Nonlinear thermal radiation and chemical reaction effects on Maxwell fluid flow with convectively heated plate in a porous medium. Heat Transf. Asian Res..

[23] Khan AA, Bukhari SR, Marin M, Ellahi R (2019). Effects of chemical reaction on third-grade MHD fluid flow under the influence of heat and mass transfer with variable reactive index. Heat Transf. Res..

[24] Mythili D, Sivaraj R (2016). Influence of higher order chemical reaction and non-uniform heat source/sink on Casson fluid flow over a vertical cone and flat plate. J. Mol. Liq..

[25] Malik YM (2016). Effects of second order chemical reaction on MHD free convection dissipative fluid flow past an inclined porous surface by way of heat generation: A Lie group analysis. Inf. Sci. Lett..

[26] Veeram G, Poojitha P, Katta H, Hemalatha S, Babu MJ, Raju CS (2022). Simulation of dissipative hybrid nanofluid (PEG-Water+ ZrO2+ MgO) flow by a curved shrinking sheet with thermal radiation and higher order chemical reaction. Mathematics.

[27] Gopal D, Saleem S, Jagadha S, Ahmad F, Almatroud AO, Kishan N (2021). Numerical analysis of higher order chemical reaction on electrically MHD nanofluid under influence of viscous dissipation. Alex. Eng. J..

[28] Acharya N, Bag R, Kundu PK (2021). Unsteady bioconvective squeezing flow with higher-order chemical reaction and second-order slip effects. Heat Transf..

[29] Kumar, M. A., Reddy, Y. D., Goud, B. S., & Rao, V. S. An impact on non-Newtonian free convective MHD Casson fluid flow past a vertical porous plate in the existence of Soret, Dufour, and chemical reaction. *Int. J. Ambient Energy*. 1–9 (2022).

[30] Ali B, Shafiq A, Siddique I, Al-Mdallal Q, Jarad F (2021). Significance of suction/injection, gravity modulation, thermal radiation, and magnetohydrodynamic on dynamics of micropolar fluid subject to an inclined sheet via finite element approach. Case Stud. Therm. Eng..

[31] Siddiqa S, Naqvi SB, Begum N, Awan SE, Hossain MA (2018). Thermal radiation therapy of biomagnetic fluid flow in the presence of localized magnetic field. Int. J. Therm. Sci..

[32] Reddy YD, Goud BS, Kumar MA (2021). Radiation and heat absorption effects on an unsteady MHD boundary layer flow along an accelerated infinite vertical plate with ramped plate temperature in the existence of slip condition. Partial Differ. Equ. Appl. Math..

[33] Ge-JiLe H, Shah NA, Mahrous YM, Sharma P, Raju CSK, Upddhya SM (2021). Radiated magnetic flow in a suspension of ferrous nanoparticles over a cone with brownian motion and thermophoresis. Case Stud. Therm. Eng..

[34] Hassan A, Hussain A, Arshad M, Alanazi MM, Zahran HY (2022). Numerical and thermal investigation of magneto-hydrodynamic hybrid nanoparticles (SWCNT-Ag) under Rosseland radiation: A prescribed wall temperature case. Nanomaterials.

[35] Hussain A, Hassan A, Al Mdallal Q, Ahmad H, Rehman A, Altanji M, Arshad M (2021). Heat transport investigation of magneto-hydrodynamics (SWCNT-MWCNT) hybrid nanofluid under the thermal radiation regime. Case Stud. Therm. Eng..

[36] Reddy YD, Goud BS, Chamkha AJ, Kumar MA (2022). Influence of radiation and viscous dissipation on MHD heat transfer Casson nanofluid flow along a nonlinear stretching surface with chemical reaction. Heat Transf..

[37] Raju CSK, Ibrahim SM, Anuradha S, Priyadharshini P (2016). Bio-convection on the nonlinear radiative flow of a Carreau fluid over a moving wedge with suction or injection. Eur. Phys. J. Plus.

[38] Sajjan K, Shah NA, Ahammad NA, Raju CSK, Kumar MD, Weera W (2022). Nonlinear Boussinesq and Rosseland approximations on 3D flow in an interruption of Ternary nanoparticles with various shapes of densities and conductivity properties. AIMS Math..

[39] Upadhya SM, Raju SSR, Raju CSK, Shah NA, Chung JD (2022). Importance of entropy generation on Casson, Micropolar and Hybrid magneto-nanofluids in a suspension of cross diffusion. Chin. J. Phys..

[40] Shankar Goud, B., Dharmendar Reddy, Y., & Mishra, S. Joule heating and thermal radiation impact on MHD boundary layer Nanofluid flow along an exponentially stretching surface with thermal stratified medium. *Proc. Inst. Mech. Eng. Part N J. Nanomater. Nanoeng. Nanosyst.* 23977914221100961 (2022).

[41] Reddy, Y. D., Mebarek-Oudina, F., Goud, B. S., & Ismail, A. I. Radiation, velocity and thermal slips effect toward MHD boundary layer flow through heat and mass transport of Williamson nanofluid with porous medium. *Arab. J. Sci. Eng.* 1–15 (2022).

[42] Animasaun IL, Sandeep N (2016). Buoyancy induced model for the flow of 36 nm alumina-water nanofluid along upper horizontal surface of a paraboloid of revolution with variable thermal conductivity and viscosity. Powder Technol..

[43] Salahuddin T, Awais M (2022). A comparative study of Cross and Carreau fluid models having variable fluid characteristics. Int. Commun. Heat Mass Transf..

[44] Abbas T, Rehman S, Shah RA, Idrees M, Qayyum M (2020). Analysis of MHD Carreau fluid flow over a stretching permeable sheet with variable viscosity and thermal conductivity. Phys. A.

[45] Khan M, Malik MY, Salahuddin T, Khan I (2017). Numerical modeling of Carreau fluid due to variable thicked surface. Results Phys..

[46] Nalivela, N. R., Vempati, S. R., Ravindra Reddy, B., & Dharmendar Reddy, Y. Viscous dissipation and thermal radiation impact on MHD mass transfer natural convective flow over a stretching sheet. *Proc. Inst. Mech. Eng. Part E J. Process Mech. Eng.* 09544089221081339 (2022).

[47] Megahed AM (2019). Carreau fluid flow due to nonlinearly stretching sheet with thermal radiation, heat flux, and variable conductivity. Appl. Math. Mech..

[48] Irfan M, Khan M, Khan WA (2018). Interaction between chemical species and generalized Fourier’s law on 3D flow of Carreau fluid with variable thermal conductivity and heat sink/source: A numerical approach. Results Phys..

[49] Waqas M, Alsaedi A, Shehzad SA, Hayat T, Asghar S (2017). Mixed convective stagnation point flow of Carreau fluid with variable properties. J. Braz. Soc. Mech. Sci. Eng..

